# Direct Photolithography of WO_x_ Nanoparticles for High-Resolution Non-Emissive Displays

**DOI:** 10.1007/s40820-024-01563-6

**Published:** 2024-11-21

**Authors:** Chang Gu, Guojian Yang, Wenxuan Wang, Aiyan Shi, Wenjuan Fang, Lei Qian, Xiaofei Hu, Ting Zhang, Chaoyu Xiang, Yu-Mo Zhang

**Affiliations:** 1 Laboratory of Optoelectronic Information Technology and Devices, Ningbo Institute of Materials Technology and Engineering, Chinese Academy of Sciences, Ningbo, 315201 People’s Republic of China; 2 Hangzhou Bay Laboratory of Advanced Nano-Optoelectronic Materials and Devices, Qianwan Institute of CNITECH, Ningbo, 315336 People’s Republic of China; 3 Smart Materials for Architecture Research Lab, Innovation Center of Yangtze River Delta, Zhejiang University, Jiaxing, 314100 People’s Republic of China; 4 University of Science and Technology of China, Hefei, 230026 People’s Republic of China; 5 State Key Lab of Supramolecular Structure and Materials, College of Chemistry, Jilin University, Changchun, 130012 People’s Republic of China

**Keywords:** Electrochromic, Direct photolithography, WO_x_ nanoparticles, In situ photo-induced ligand exchange, High-resolution displays

## Abstract

High-resolution non-emissive displays based on electrochromic tungsten oxides (WO_x_) are crucial for future near-eye virtual/augmented reality interactions, given their impressive attributes such as high environmental stability, ideal outdoor readability, and low energy consumption. However, the limited intrinsic structure of inorganic materials has presented a significant challenge in achieving precise patterning/pixelation at the micron scale. Here, we successfully developed the direct photolithography for WO_x_ nanoparticles based on in situ photo-induced ligand exchange. This strategy enabled us to achieve ultra-high resolution efficiently (line width < 4 µm, the best resolution for reported inorganic electrochromic materials). Additionally, the resulting device exhibited impressive electrochromic performance, such as fast response (< 1 s at 0 V), high coloration efficiency (119.5 cm^2^ C^−1^), good optical modulation (55.9%), and durability (> 3600 cycles), as well as promising applications in electronic logos, pixelated displays, flexible electronics, etc. The success and advancements presented here are expected to inspire and accelerate research and development (R&D) in high-resolution non-emissive displays and other ultra-fine micro-electronics.

**Supplementary Information:**

The online version contains supplementary material available at 10.1007/s40820-024-01563-6.

## Highlights

- Direct photolithography of electrochromic WO_x_ nanoparticles via in situ photo-induced ligand exchange is proposed and demonstrated.
- The highest resolution among inorganic electrochromics (< 4 µm) is achieved, which is promising in high-resolution non-emissive displays.
- The as-prepared device exhibits highly remarkable performance including rapid response, high coloration efficiency and durability.

**Supplementary Information:**

The online version contains supplementary material available at 10.1007/s40820-024-01563-6.

## Graphical abstract

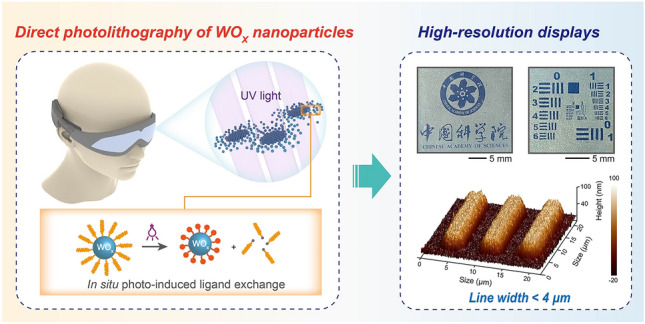

## Introduction

Emerging electrochromic (EC) materials and devices, capable of efficiently and reversibly modulating optical signals (color, absorbance, transmittance, reflectivity, etc.) and information through electrochemically driven redox processes, offer compelling features such as outdoor readability, flexibility, transparency, and low energy consumption [1–6]. These impressive properties have positioned EC materials and devices as pivotal components in wearable electronics [7–9], reflective/transparent non-emissive displays [10–12], and other applications. Given the escalating demand for higher image quality and broader application scenarios (e.g., near-eye virtual/augmented reality displays [13, 14], display-on-a-chip [15], etc.), achieving ultra-high resolution with ideal EC performance is crucial for the advancement of EC display technologies.

Inorganic EC materials, particularly transition metal oxides exemplified by tungsten oxide (WO_x_, 0 < x ≤ 3) [16–20], have attracted significant attention in EC applications like smart windows, sunroofs, and electronic shelf labels, owing to their compelling durability and stability, which are also essential for the current displays to adapt to various environments. Recent advancements in WO_x_ nanomaterials and feasible solution-processing techniques have demonstrated superior EC performance and cost-effectiveness compared to traditional WO_x_ films produced by magnetron sputtering [21–25]. Furthermore, classic processing strategies (e.g., inkjet printing, dispensing printing, etc.) have been proposed and made positive progress in preparing WO_x_ patterns to meet the requirements of EC displays [26–28]. For instance, P. Yang, T. Kraus, and H. J. Fan et al. reported a flexible pseudocapacitive EC device based on inkjet-printed WO_3-x_ nanoparticles (NPs) [29]. However, due to nozzle limitations and the ever-present coffee-ring effect, these patterning strategies have so far achieved only relatively low resolutions (≥ 20 µm) with inevitable nonuniformity issues. High-resolution patterning/pixelation of WO_x_ nanomaterials at the micron scale remains a challenging objective. Further R&D efforts are urgently required to address these challenges and unlock the full potential of high-resolution EC displays.

Direct photolithography of functional materials, utilizing photo-induced chemical reactions to modify material solubility, is an emerging and highly anticipated strategy for high-resolution, high-throughput and high-fidelity patterns [30–36]. For example, Wang and Talapin et al. reported the the photo-patterning of inorganic nanomaterials based on in situ ligand exchange between nanomaterials and photoacid generators (PAGs) [33–36]. In the field of EC displays, direct photolithography of organic materials has been reported many times due to their excellent structural modifiability and rich colors. For example, John R. Reynolds et al. demonstrated the first direct photo-patterning of EC conjugated polymers (CPs) in 2008 [37], with subsequent reports achieving resolutions of approximately 50 µm for photolithographic CPs [10, 38–40]. Additionally, J.-M. Myoung et al. reported multi-color pixelated gels (size = 200 µm) based on viologen derivatives (organic EC molecules) [41, 42]. In our previous work, the direct photolithography of EC dyes was demonstrated, achieving an outstanding resolution (~ 2 µm) and EC performance based on proton-coupled electron transfer mechanism [43, 44]. The aforementioned results highlight the value of direct photolithography for organic EC materials. However, the unsatisfactory environmental stability of organic materials, which could not be ignored, limits their practical applications [5]. In contrast, directly photolithographic EC WO_x_ could be a promising way to simultaneously achieve the excellent stability and high resolution. But unfortunately, due to its poor modifiability stemming from intrinsic structural limitations, this goal has not been realized. Besides, the relatively low coloration efficiency and response speed of inorganic materials also limit their potential application and development in the field of high-resolution display to a certain extent.

Herein, a novel high-resolution EC system based on WO_x_ NPs and photosensitive additives was introduced to address the above bottleneck. And precise WO_x_ patterns (line width < 4 µm, one of the highest resolutions in the EC field) could be achieved successfully through a well-designed photolithographic process involving in situ ligand exchange reactions under UV radiation. Meanwhile, the as-prepared device exhibited remarkable EC performance, including rapid response, high coloration efficiency, good optical modulation and durability. Furthermore, some non-emissive display prototypes with potential applications in self-powered displays, electronic logos, pixelated displays, and flexible electronics, were demonstrated, showcasing its wide applicability. The interesting investigation about direct photolithography of EC WO_x_ NPs represented here makes a significant advancement in the development of high-resolution non-emissive EC displays and other ultra-fine micro-electronics. And the promising potential of this work extends to applications in academic and research communities.

## Experimental Section

### Synthesis of WO_x_ NPs

**Figure 1:**
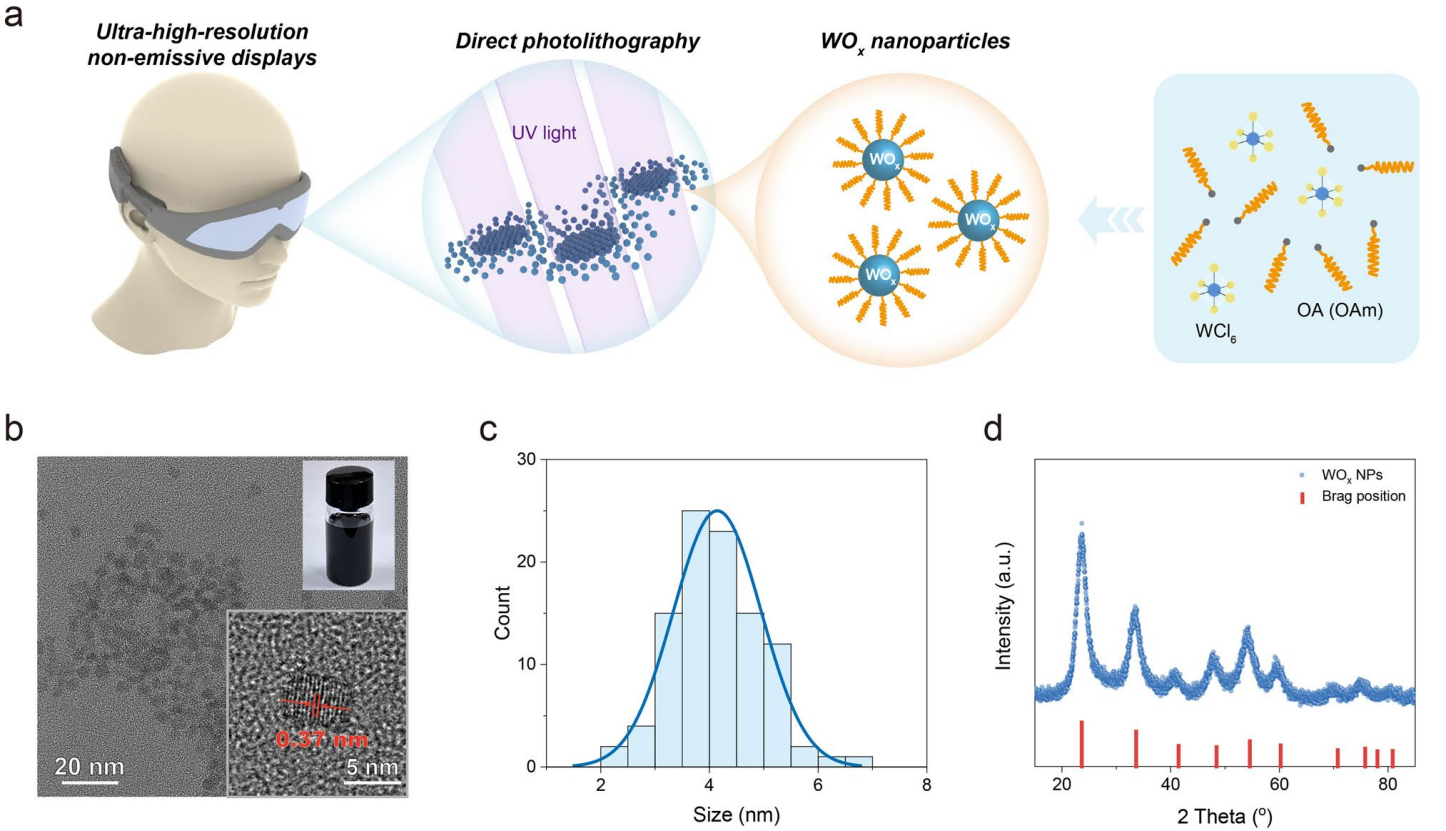
Synthesis of WO_x_ NPs. **a** Schematic of direct photolithography of WO_x_ NPs synthesized by WCl_6_ and OA (and/or OAm) for ultra-high-resolution EC non-emissive displays, **b** TEM image of WO_x_ NPs at different magnifications. Insert picture: the photo of toluene solution containing WO_x_ NPs, **c** Diameter statistics of as-synthesized WO_x_ NPs, **d** X-ray diffraction pattern of WO_x_ NPs. Red bars: Brag positions of the cubic WO_3_ phase (JCPDS 46–1096)

WO_x_ NPs were synthesized according to the reported method with some modifications [29]. At first, tungsten chloride (WCl_6_) powder (800 mg) was dissolved in oleic acid (8 mL) under stirring in a nitrogen-filled glove box. Meanwhile, oleylamine (OAm, 4 mL) and oleic acid (OA, 40 mL) were placed into a dried round bottom flask. The mixture solution was degassed at 120 °C for 1 h, and then was heated up to 300 °C under Argon flow. The precursor of WCl_6_ was injected into the abovementioned solution quickly and maintained for 5 min. After that the reaction system was cooled down to room temperature, the raw WO_x_ nanoparticles were obtained by precipitation into excessive isopropanol and centrifugation. At last, WO_x_ NPs were washed twice by isopropanol and dispersed in a nonpolar solvent (e.g., toluene, or n-hexane, or their mixed solution). The photo of as-prepared solution is listed in the insert picture in Fig. 1b. The concentration of WO_x_ NPs was approximately 30 mg mL^−1^.

### Direct Photolithography of WO_x_ NPs

In order to achieve direct photolithography of WO_x_ NPs, a transparent electrode (ITO or PET-ITO) was used as the substrate. A WO_x_ film was coated on the substrate by spin coating using the photolithographic solution (30 mg mL^−1^ WO_x_ in toluene, or n-hexane, or their mixed solution with 2-(4-methoxystyryl)-4,6-bis(trichloromethyl)-1,3,5-triazine (MBT)). Unless otherwise mentioned, the content of MBT was controlled to 1.0 wt% of WO_x_. The WO_x_ film was exposed under UV LED lamp (centered at 365 nm) with different photomasks for required patterns in a nitrogen-filled glove box. Then, the film was submerged in toluene to wash off the unexposed areas (as the developing process). At last, the film was annealed at 100 °C for 20 min to completely remove the residual solvent. The thickness of as-prepared WO_x_ film and pattern could be adjusted by controlling the spinning speed, as shown in Table S1.

### Preparation and Characterization of EC Devices

In order to prepare an EC device, at first, the direct photolithography of WO_x_ on ITO (or PET-ITO for flexible devices) was developed as the EC electrode. And then, a PDMS spacer (thickness: ~ 390 μm) was prepared. A Zn foil (thickness: ~ 120 μm) was carved into the appropriate shape. At last, the device was successfully fabricated by combining the EC electrode, a PDMS spacer, an electrolyte solution (1.0 mol L^−1^ ZnSO_4_ in deionized water), a Zn foil and a transparent glass cover. EC properties of as-prepared devices were characterized by a UV–Vis spectrophotometer integrated with an electrochemical workstation. Air was used as the reference.

## Results and Discussion

### Synthesis of EC WO_x_

In order to efficiently fabricate high-resolution WO_x_-based EC displays using the direct photolithographic strategy, as shown in Fig. 1a, the key lies in constructing a highly compatible combination of WO_x_ and photosensitive additive. The photosensitive additive, can serve as an effective dissolution inhibitor for the formation of WO_x_ patterns under suitable UV radiation. And WO_x_ serves as the basis for EC function, which is the prerequisite for application. Therefore, the design and synthesis of WO_x_ were the initial focus of this research.

WO_x_ NPs were selected as the EC material due to their large specific surface area, good dispersibility, and ideal film-forming properties [45]. Herein, WO_x_ NPs were synthesized using a classic solvothermal process with WCl_6_ as the W source (Fig. 1a), resulting in a dark blue solution (insert picture in Fig. 1b). Transmission electron microscope (TEM) images revealed clear lattice fringes with a spacing of 0.37 nm in the prepared WO_x_ NPs, corresponding to the (200) crystal face. As shown in Fig. 1c, the size distribution of WO_x_ NPs was in a relatively narrow range of 2–7 nm in diameter (average diameter: 4.14 nm), indicating excellent uniformity. The elemental mapping in aberration corrected scanning transmission electron microscopy (STEM) using energy-dispersive X-ray spectra (EDS) confirmed the presence of W and O elements (Fig. S1). Furthermore, the crystalline phase of as-synthesized WOx NPs was determined as a cubic phase by X-ray diffraction (XRD) analysis (Fig. 1d). Meanwhile, the calculated size of WO_x_ NPs was about 4.20 nm based on Scherrer equation (Note S3), which proved the accuracy of diameter statistics based on TEM data.

Considering the volatile feature of organic components, the content of surface organic ligands could be measured by thermogravimetric analysis (TGA) [46]. Here, the weight ratio of WO_x_ NPs was maintained at 88.3% after 450 °C, proving that its content of organic ligands (OA and/or OAm) was ~ 11.7% (Fig. S2). Based on these experimental results, WO_x_ NPs dispersed in a nonpolar solvent (e.g., toluene or n-hexane) were successfully prepared for further EC research and applications.

### Direct Photolithography of As-Synthesized WO_x_ NPs

**Figure 2:**
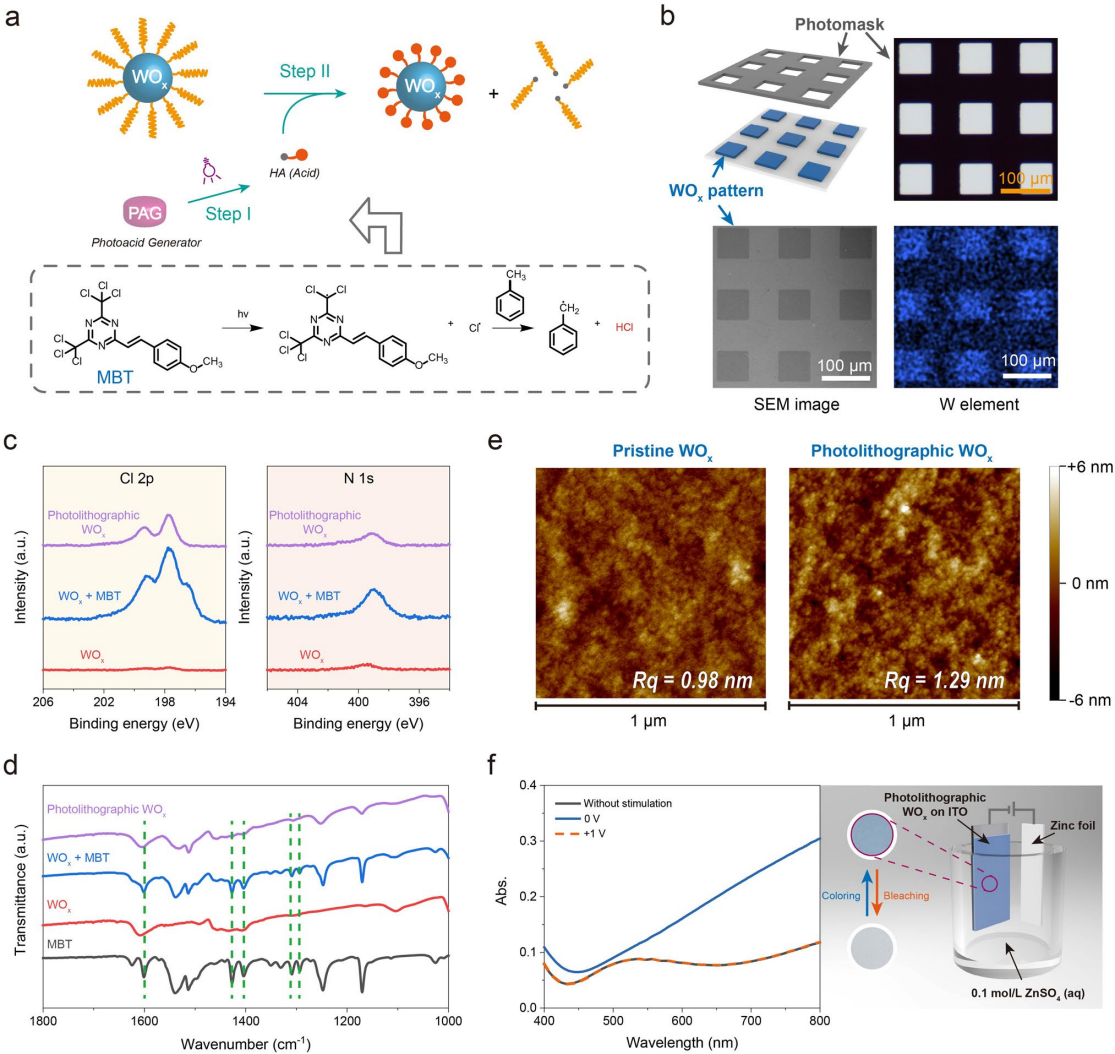
Direct photolithography of WO_x_ NPs. **a** Top: schematic of direct photolithography of WO_x_ NPs via in situ ligand exchange. Bottom: the photochemical reaction of MBT, **b** Top: schematic and microscopic image of the photomask. Bottom: SEM image and SEM–EDS elemental mapping (W) of as-prepared photolithographic WO_x_ pattern, **c** Cl 2*p* (left) and N 1*s* (right) spectra of pristine WO_x_, WO_x_ with 20.0 wt% MBT, and photolithographic WO_x_. Related intensity data were normalized with the intensity of W 4*f*. A residual signal of N 1*s* was still observed in photolithographic WO_x_ possibly caused by the introduction of excess MBT, **d** FT-IR spectra of MBT, WO_x_, WO_x_ with 20.0 wt% MBT, and photolithographic WO_x_. A transparent KBr wafer was used as the substrate, **e** AFM images of pristine WO_x_ and photolithographic WO_x_, **f** Left: absorption spectra in visible region of photolithographic WO_x_ on ITO under different electrochemical stimulations. Right: photos of photolithographic WO_x_ film (diameter: 1 cm) and schematic of electrochemical measurement. 0.1 mol/L ZnSO_4_ in deionized water was used as the electrolyte solution

Finding a suitable photosensitive additive with high photosensitivity, outstanding reactivity, and compatibility is crucial for achieving precise optical patterning of as-prepared EC materials (WO_x_ NPs). Here, PAGs are considered ideal candidates. In this expected process, PAGs release protons (H^+^) under UV radiation, as shown in Fig. 2a. These protons can react with the surface organic ligands of WO_x_ NPs, leading to an in situ ligand exchange process. The solubility of WO_x_ NPs with new ligands in non-polar solvents decrease significantly. Consequently, various patterns can be easily achieved. Herein, MBT was selected as the PAG due to its strong absorption in UVA region (320–400 nm) (Fig. S3) and good solubility in toluene (the dispersion solvent for WO_x_ NPs). Moreover, MBT exhibited high photosensitivity to UV radiation. As demonstrated in Fig. S3, the intensity of its characteristic absorption peak decreased significantly upon radiation with i-line (365 nm, industrial-standard light sources for photolithography). The photochemical reaction mechanism of MBT was illustrated in Fig. 2a, as reported previously [35].

To verify the directly photolithographic feasibility, the solubility of WO_x_ NPs with and without MBT under UV radiation was monitored (Fig. S4). Results showed that some insoluble precipitates appeared in the dispersion of WO_x_ NPs with MBT after UV radiation for 20 s. In contrast, the pristine dispersion of WO_x_ NPs remained stable, indicating that WO_x_ NPs and MBT could be an efficient candidate for direct optical patterning. As a proof of concept, a high-resolution array was achieved on a transparent conductive electrode (indium tin oxide, ITO) after UV radiation and developing processes. And the uniform distribution of tungsten (W) element, as shown in Fig. 2b, indicated that the array was composed of WO_x_.

Further, to elucidate the light-triggered in situ ligand exchange mechanism, detailed changes in component structure before and after the photolithographic process were systematically studied. As shown in Figs. 2c and S5, apparent characteristic peaks of MBT (Cl 2*p* and N 1*s*) were observed in the film containing WO_x_ and MBT, as monitored by X-ray photoelectron spectroscopy (XPS) analysis. It is noteworthy that the MBT content was set at 20.0 wt% to amplify its signal, which was significantly higher than the default content (1.0 wt%). After the UV radiation and developing process, the characteristic peak of Cl 2*p* was well maintained, verifying the successful ligand exchange. Related mechanism was further confirmed by Fourier transform infrared (FT-IR) spectra (Fig. 2d). Similar to XPS analysis, characteristic peaks of MBT were detected in the film containing WO_x_ and MBT. On the other hand, these peaks became very weak after the photolithographic process, demonstrating the effective removal of most residual groups of MBT.

Then, the morphology differences of pristine and photolithographic WO_x_ were explored by atomic force microscope (AFM) analysis. Results showed that the root-mean-square roughness (*R*_q_) value of the pristine WO_x_ film was only 0.98 nm (Fig. 2e), which proved its good film-forming ability. And the Rq value of the photolithographic WO_x_ film was 1.29 nm. The slight increase in roughness might be attributed to the surface ligand exchange of WO_x_ NPs, which was consistent with the elemental and spectral analysis (SEM–EDS, XPS, and FT-IR). According to the above experimental results, the mechanism of the photolithography process is described in Fig. S6, which is in line with the design concept shown in Fig. 2a. On the other hand, both pristine and photolithographic WO_x_ films showed strong adhesion with ITO electrode (Note S4 and Fig. S8). And related classification reaches the highest level (5B) according to the international cross-cut method of Standard Test Methods for Rating Adhesion by Tape Test in ASTM-3359 (ASTM, American Society for Testing and Materials).

Importantly, the photolithographic WO_x_ film demonstrated a satisfying ability to adjust the visible spectrum and color. As illustrated in Fig. 2f, it could switch reversibly between a colorless state and a blue-colored state when external voltages were applied (0 V vs. Zn/Zn^2+^ for the coloring process, +1 V vs. Zn/Zn^2+^ for the bleaching process), similar to the pristine WO_x_ film (Fig. S9). Note that the optical modulation of photolithographic WO_x_ film was higher than pristine WO_x_ film under the same stimulation. We hypothesize that the underlying reasons might be: (1) the improved conductivity due to the ligand exchange that replacing long-chain organic ligands to Cl; (2) the increased roughness (as shown in Fig. 2e), which would increase reactive sites and transfer rates of ions. Cyclic voltammetry (CV) tests of as-prepared pristine WO_x_ and photolithographic WO_x_ were performed to support this hypothesis. Results showed that both of them had similar electrochemical reduction and re-oxidation signals (Fig. S10), proving that they underwent the same reaction process. Moreover, the current density of photolithographic WO_x_ was slightly higher, which supported the improvement of conductivity after ligand exchange. In a word, the promising EC property of photolithographic WO_x_ fully proved its potential for deeper application in EC displays.

### Fabrication and Optimization of Self-Powered EC Devices

**Figure 3:**
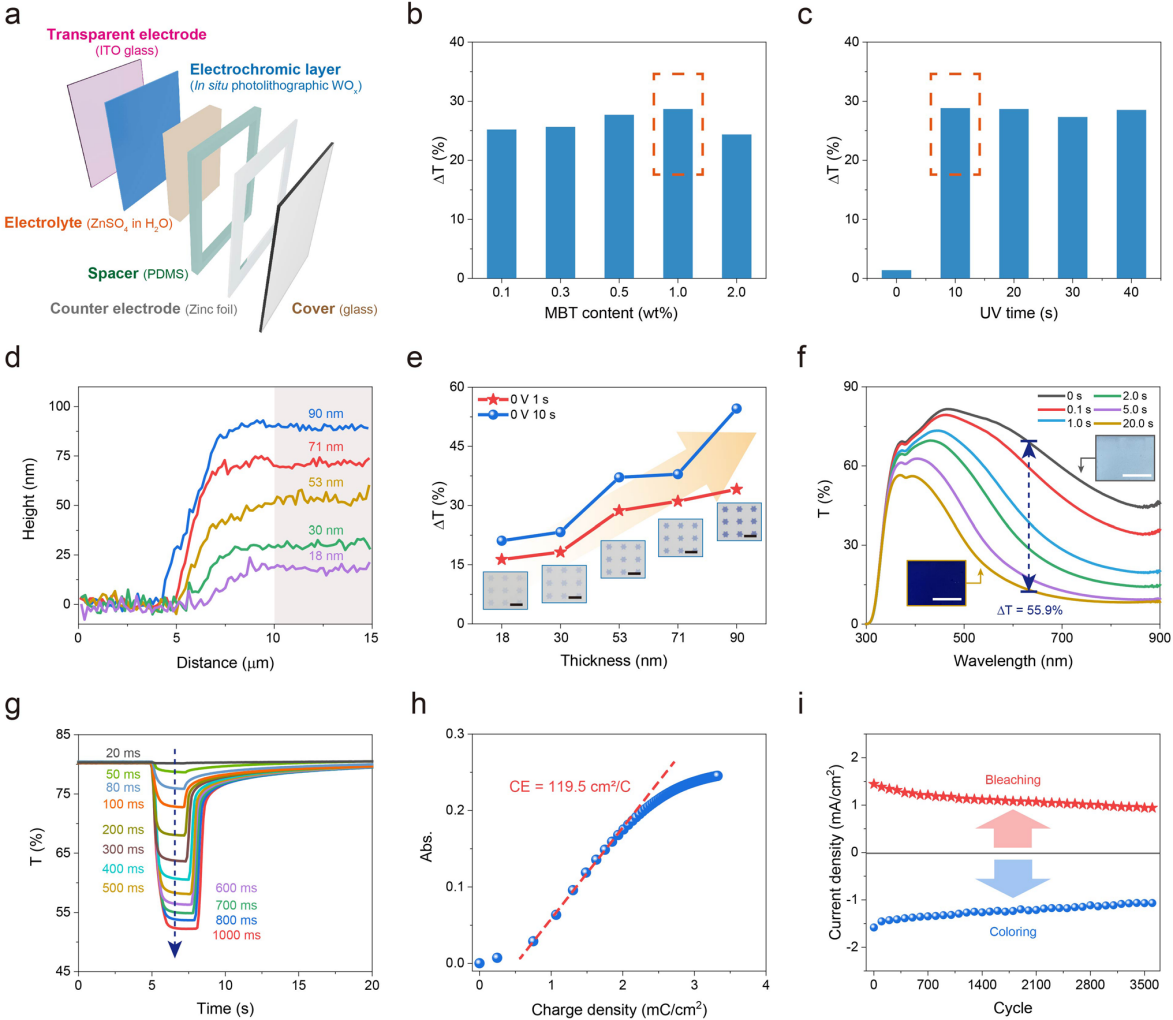
Fabrication and optimization of self-powered EC devices. **a** Schematic of the self-powered EC device, **b** Optical modulation (ΔT) at 633 nm of EC devices with different MBT contents in their photolithographic processes, **c** ΔT at 633 nm of EC devices with different UV radiation times in their photolithographic processes. 0 V 1 s was applied for the coloring process, **d** Height profiles of the edge of photolithographic WO_x_ films, **e** ΔT at 633 nm of as-prepared EC devices based on photolithographic WO_x_ films with different thickness. 0 V 1 s and 10 s were applied for coloring processes, respectively. Insert: photos of as-prepared photolithographic WO_x_ patterns in their colored states. Scale bar: 100 μm, **f** UV–vis spectra of EC device with photolithographic WO_x_ film (thickness: ~ 90 nm) under electrical stimulations of 0 V for different times. Insert: photos of the device in its colorless state and colored state. Scale bar: 1 cm, **g** Transmittance (T%) at 633 nm of the EC device under 0 V for different times in coloring processes. +1 V was used for bleaching processes, **h** CE at 633 nm of the device under 0 V, **i** The peak current density of the device in the coloring process and bleaching process at different cycles in CV tests

Based on the directly photolithographic WO_x_ film on transparent ITO electrode (as the EC electrode), an EC device was fabricated by assembling the EC electrode, an electrolyte solution (1.0 mol L^−1^ ZnSO_4_ in deionized water), a patterned PDMS spacer, a counter electrode (a carved Zn foil), and a transparent glass cover (Fig. 3a). As shown in Fig. S11, the device could switch from a transparent colorless state to blue at 0 V due to the low potential of Zn/Zn^2+^. Meanwhile, the device could also revert to the original transparent colorless state spontaneously. It means that the device operates without the supply of energy and is known as a self-powered EC device [47–50]. Moreover, a positive voltage (+1 V) could accelerate the bleaching process and is therefore used for subsequent experiments.

Further, to gain a deeper understanding of this new EC system, we investigated the effects of preparation parameters on photolithographic resolution and EC property. Herein, MBT content and UV radiation dose (time) were analyzed. As illustrated in Fig. S12, a hexagram array (size: 50 μm) with a clear edge could be obtained, even when the MBT content was as low as 0.1 wt%. On the other hand, related devices exhibited slightly different EC performance, as shown in Fig. 3b. Meanwhile, the background color of all devices was very weak, which could be verified from their transmittance spectra in the colorless state (Fig. S14). Considering the highest optical modulation when MBT content was 1.0 wt%, this was considered the optimal parameter for reference in subsequent experiments. Similarly, when the UV radiation time reached 10 s (approximately 600 mJ cm^−2^), a high-resolution WO_x_ pattern with promising EC properties could be efficiently formed (Figs. 3c and S13).

The thickness of photolithographic WO_x_ films could be dynamically controlled from ~ 18 to ~ 90 nm by varying spin speeds (Fig. 3d and Table S1). Remarkably, as the thickness increased, the resolution of direct photolithography was well maintained, as shown in the inset photos of Fig. 3e. Meanwhile, their optical modulation (ΔT) increased, with the maximum reaching 55.9% (Fig. 3e, f). And it is promising to achieve better optical modulation through thicker EC films, which is preferred in the field of non-emissive EC displays. However, achieving ultra-high resolution on a very thick film is still a challenging, limited by the aspect ratio of photolithography. Meanwhile, the increase of film thickness will also affect ion transfer process, resulting in slow response speed. In addition, compared to organic dyes or conjugated polymers, the lower coloration efficiency of inorganic EC materials also limits their optical contrast. In the future, the solution of above bottlenecks is expected to further accelerate the development of inorganic EC displays. Gratifyingly, in this work, a typical EC device (thickness of photolithographic WO_x_ film: ~ 53 nm) exhibited attractive optical modulation (28.7% at 0 V 1 s, and 37.1% at 0 V 10 s). Its UV–vis spectra and photos at different optical states are listed in Fig. S15.

This device displayed a gradient variation of transmittance regulated with the time of applied voltage ranging from 20 to 1000 ms (Fig. 3g). Results revealed that only a short response time of 50 ms was required to achieve a significant change in transmittance. It is worth noting that because shapes of EC electrode and counter electrode do not perfectly overlap, the effective ion transport distance is longer than the thickness of the electrolyte layer. Therefore, the true response time of EC materials (WO_x_ NPs) will be shorter than the measured one. Additionally, the coloration efficiency (CE) of the device was approximately 119.5 cm^2^ C^−1^, indicating a high electronic utilization efficiency (Fig. 3h).

Cycling stability is an essential parameter for evaluating application potentials of EC devices and displays [5]. Here, the device based on the photolithographic WO_x_ film maintained its electrochemical reduction and re-oxidation performance after 3600 cycles in CV tests between 0 V and +1 V (Figs. 3i and S16). During this process, the device underwent complete coloring and bleaching processes, as shown in Fig. S16. Gratifyingly, the EC performance, represented by optical modulation, was well maintained after the aforementioned CV tests (Fig. S17). The residual optical modulation was about 22.4%, and its maintenance ratio reached ~ 78%, indicating the good durability. Compared with reported devices based on WO_x_ (Table S2), the EC device we developed exhibits promising color-switching performance and holds application value in EC displays.

### Applications of High-Resolution EC Displays

**Figure 4:**
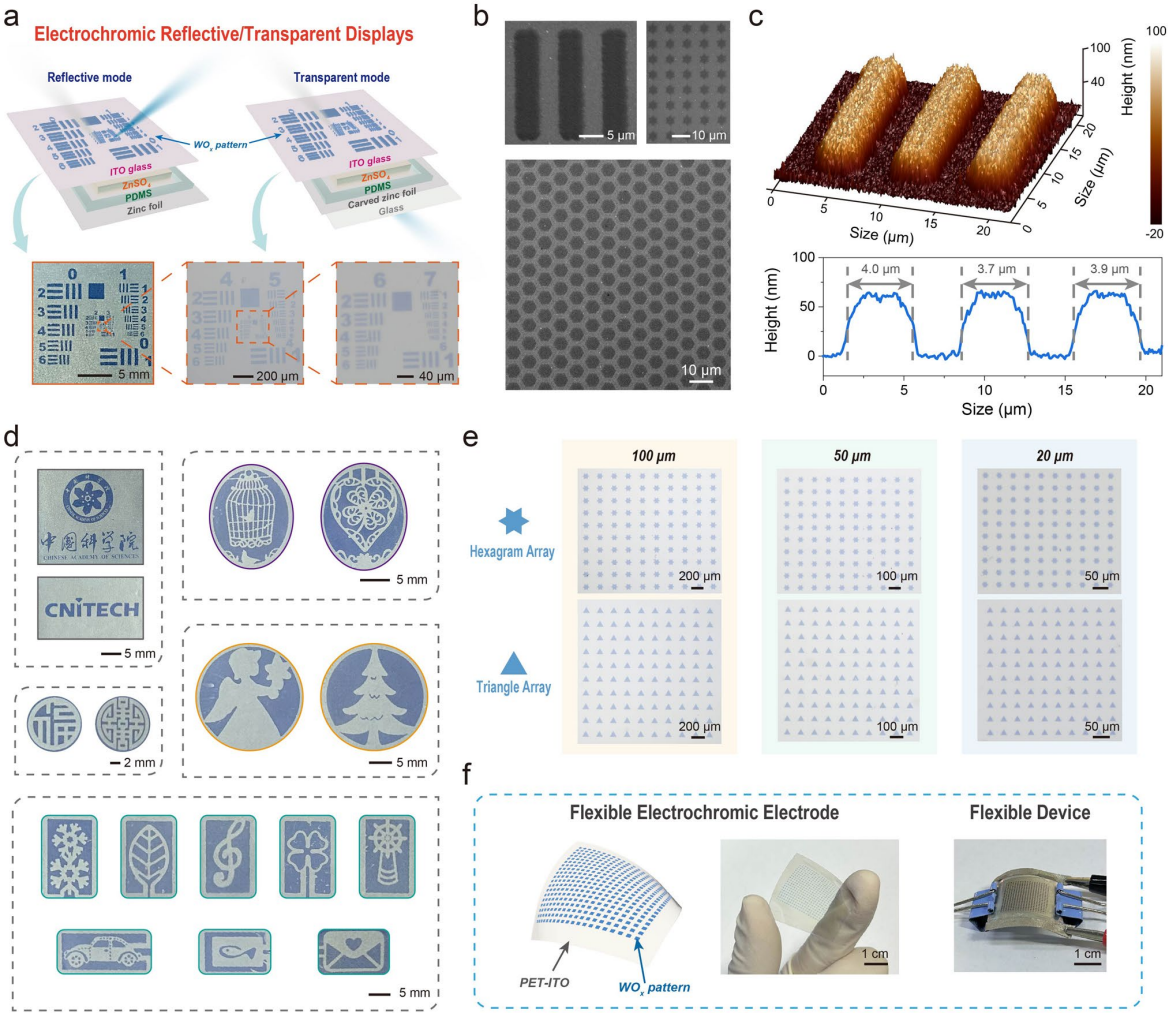
Applications of high-resolution EC devices. **a** Schematic and related photos of as-prepared EC reflective and transparent displays. Microscopic images were taken by transparent displays, **b** SEM images of EC patterns based on photolithographic WO_x_ NPs, **c** Three-dimensional AFM image and corresponding surface profile of photolithographic EC patterns, **d** Photos of EC reflective displays with different photolithographic patterns, **e** Microscopic images of EC transparent displays with different photolithographic arrays, **f** Schematic and photos of as-prepared flexible EC electrode and device

In order to validate the application potential of photolithographic WO_x_ NPs in high-resolution reflective displays and transparent displays (Fig. 4a), various EC WO_x_ devices were fabricated successfully. Firstly, the resolution of EC patterns was determined. According to corresponding 1951 USAF resolution microscopic images (Fig. 4a), SEM data (Fig. 4b), and AFM data (Fig. 4c), the minimum pattern size (line width) could reach below 4 μm. To our knowledge, this is the highest resolution for inorganic EC materials/device. Meanwhile, this still falls within the highest range even compared with reported organic EC patterns (Fig. S18 and Table S3). This means that the direct photolithography of inorganic EC materials, as well as the related WO_x_ NPs-based devices, is suitable for future high-resolution display scenes, for example, near-eye augmented reality and virtual reality.

Then, we prepared a series of WO_x_ patterns and constructed corresponding EC devices using various photomasks (Fig. 4d). These patterned devices could be utilized in daily decorations, electronic logos, EC signs, and other display applications. Importantly, we developed EC devices with high-resolution hexagram/triangle pixels arrays (pixel size = 100, 50, and 20 μm, respectively) (Fig. 4e), laying the foundation for future active-matrix (AM) and/or passive-matrix (PM) driven pixelated displays. Subsequently, we will explore the application potential of WO_x_ NPs in high-resolution pixelated displays based on as-developed photolithographic patterns. In this process, the driving voltage of EC pixels and the signal crosstalk between EC pixels, as well as the addressing/driven mode of the external circuit, will be studied in detail.

Additionally, direct photolithography of WO_x_ NPs was performed on a flexible conductive substrate (polyethylene terephthalate-coated ITO, PET-ITO), as shown in Fig. 4f. Subsequently, a flexible reflective device was successfully fabricated. This flexible device maintained its EC ability even at a highly bent state (radius of curvature = 4 cm). Based on the above exploration, this newly developed EC display not only demonstrates potential in current application scenarios but also aligns with the future direction of high-resolution and flexible electronics development.

Thanks to the remarkable resolution and self-powered EC features, directly photolithographic WO_x_ NPs might also be expected to have important R&D value in the field of microelectronics (e.g., micro-nano grating, micro-iris, micro-robots, micro-batteries). As a simple proof of concept, energy storage properties of an as-prepared EC device were tested. Results revealed that the device showed typical pseudocapacitive features with 2.56 mF cm^−2^ at 0.1 mA cm^−2^ (Fig. S19). Moreover, its charging and discharging processes were accompanied by apparent transmittance changes, corresponding to the coloring and bleaching processes. This indicated that the visual energy storage was realized successfully. In addition, after 2000 charge/discharge cycles, its capacity could still be maintained at more than 84.8% of the initial one (Fig. S20), proving excellent stability of the device.

### Extendibility of Direct Photolithography Based on WO_x_ NPs

**Figure 5:**
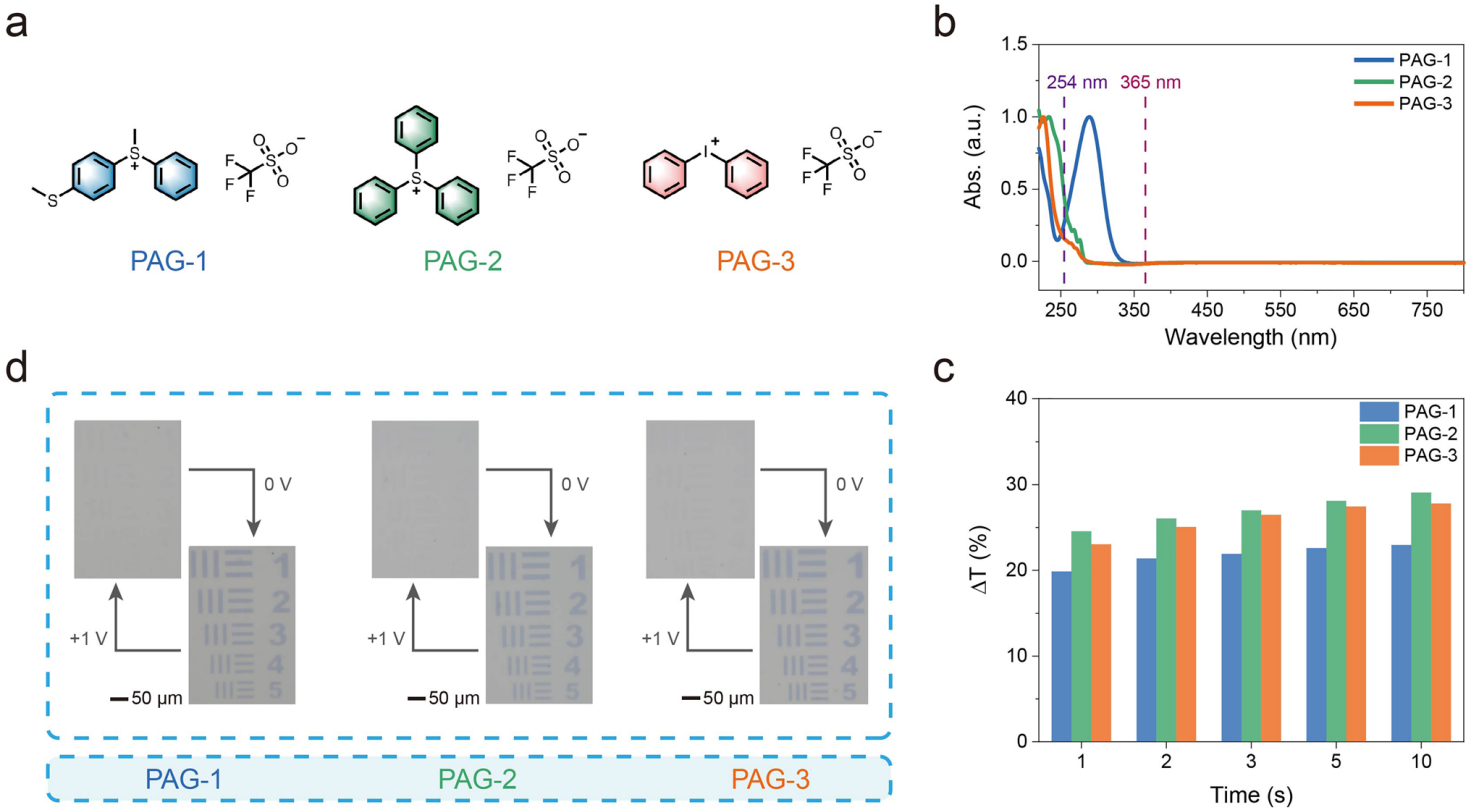
Extendibility of direct photolithography of WO_x_ NPs. **a** Molecular formula of different PAGs, **b** The UV–vis spectra of PAGs (0.01 mg/mL in ethanol), **c** ΔT at 633 nm of EC devices based on photolithographic WO_x_ with different PAGs. 0 V for different times (1, 2, 3, 5, and 10 s) were used for coloring processes, **d** Photos of as-prepared EC devices

Benefiting from the facile and efficient preparation strategy of directly photolithographic WO_x_ NPs, the developed EC system was easily expanded by changing the type of chosen photosensitive additives (PAGs). As illustrated in Fig. 5a, three other PAGs (PAG-1: (4-methylthiophenyl)methyl phenyl sulfonium triflate, PAG-2: triphenylsulfonium trifluoromethanesulfonate, PAG-3: diphenyliodonium triflate) were introduced. Since these PAGs had no absorption to i-line (365 nm) (Fig. 5b), efficient deep UV radiation (centered at 254 nm, 20 s) was used to achieve the required photolithographic process. The photochemical reaction of these PAGs was shown in Fig. S21. As expected, high-resolution WO_x_ patterns and corresponding EC devices could also be fabricated. These patterned EC devices also exhibited reversible color-switching properties, similar to the photolithographic EC system based on MBT, as revealed in Fig. 5c, d. These successful results indicate that high-resolution WO_x_ patterns can be prepared with different PAGs, which confirms the extendibility of photolithographic WO_x_ systems.

At the same time, we note that EC performance of these devices is not completely consistent, which might be due to the differences in photosensitivity of PAGs or exchanged ligands (e.g., chloride, triflate). This phenomenon suggests that in the future, the performance of devices can be dynamically adjusted through targeted optimization of the structure of photosensitive materials. This will be beneficial for further performance modifications to be suitable in various application situations.

## Conclusions

We have successfully developed a direct photolithography strategy for WO_x_ NPs to enable high-resolution EC displays, utilizing light-triggered ligand exchange. The photolithographic process was thoroughly investigated through XPS, FT-IR, and AFM analyses. The resulting EC patterns and devices exhibited ultra-high resolution (line width < 4 µm) and impressive overall EC performance, including fast response (< 1 s at 0 V), high coloration efficiency (119.5 cm^2^ C^−1^), good optical modulation (55.9%), and durability (> 3600 cycles). Furthermore, we have demonstrated promising display prototypes, showcasing the potential of practical applications of this strategy.

This innovative strategy, along with the associated materials and devices, holds great promise in paving the way for widespread applications of ultra-fine EC and other information displays. The advancements made in this work are expected to catalyze further R&D of next-generation micro-electronic technologies, ultimately contributing to the realization of advanced and versatile information display systems.

## Supplementary Information

Below is the link to the electronic supplementary material.

Supplementary file1 (DOCX 1775 KB)
