# Supplementary material for: Direct Photolithography of WOx Nanoparticles for High-Resolution Non-Emissive Displays
Source: Nanomicro Lett. 2024 Nov 21;17:67. doi: 10.1007/s40820-024-01563-6 (PMC11579263; doi:10.1007/s40820-024-01563-6)
Supplement: Supplementary file 1 — Supplementary file1 (DOCX 1775 KB) [file 40820_2024_1563_MOESM1_ESM.docx]

Supporting Information for

Direct Photolithography of WO_x_ Nanoparticles for High-Resolution Non-Emissive Displays

Chang Gu^1,2^, Guojian Yang^1,3,^*, Wenxuan Wang^1,4^, Aiyan Shi^1,3^, Wenjuan Fang^1,2^, Lei Qian^1,2^, Xiaofei Hu^1,2^, Ting Zhang^1,2,^*, Chaoyu Xiang^1,2,^* and Yu-Mo Zhang^5,^*

^1^ Laboratory of Optoelectronic Information Technology and Devices, Ningbo Institute of Materials Technology and Engineering, Chinese Academy of Sciences, Ningbo, Zhejiang 315201, P. R. China

^2^ Hangzhou Bay Laboratory of Advanced Nano-Optoelectronic Materials and Devices, Qianwan Institute of CNITECH, Ningbo, Zhejiang 315336, P. R. China

^3^ Smart Materials for Architecture Research Lab, Innovation Center of Yangtze River Delta, Zhejiang University, Jiaxing 314100, P. R. China

^4^ University of Science and Technology of China, Hefei 230026, P. R. China

^5^ State Key Lab of Supramolecular Structure and Materials, College of Chemistry, Jilin University, Changchun 130012, P.R. China

* Corresponding authors. E-mail: [yangguojian1@nimte.ac.cn](mailto:yangguojian1@nimte.ac.cn) (Guojian Yang); [zhangting@nimte.ac.cn](mailto:zhangting@nimte.ac.cn) (Ting Zhang); [xiangchaoyu@nimte.ac.cn](mailto:xiangchaoyu@nimte.ac.cn) (Chaoyu Xiang); [zhangyumo@jlu.edu.cn](mailto:zhangyumo@jlu.edu.cn) (Yu-Mo Zhang)

# Supplementary Notes

## Note S1 Materials

Tungsten chloride (WCl_6_, 99.5%) was obtained from Energy Chemical (3A) Company. Zinc sulfate heptahydrate (ZnSO_4_·7H_2_O, 99%) was purchased from Macklin Company. Oleic acid (OA, 90%) was purchased from Alfa Aesar. Oleylamine (OAm, C18: 80-90%) and n-hexane (≥98%) were purchased from Aladdin Reagent Company. Toluene and isopropanol were obtained from Sinopharm Chemical Reagent Co., Ltd. PAGs including MBT (2-(4-methoxystyryl)-4,6-bis(trichloromethyl)-1,3,5-triazine, 98%), PAG-1 ((4-methylthiophenyl)methyl phenyl sulfonium triflate), PAG-2 (triphenylsulfonium trifluoromethanesulfonate) and PAG-3 (diphenyliodonium triflate, ≥99%) were obtained from Sigma Aldrich. Zn foil (>99.9%) was purchased from Yuqian Metal Materials, Hebei province, China.

ITO (10 Ω sq^-1^) and PET-ITO (15 Ω sq^-1^) were obtained from HUAYI ELECTRONIC Co., Ltd, Southern China Xiang's Science & Technology, and Liaoning Advanced Election Technology, respectively. ITO was cleaned with deionized water, and then washed with acetone and isopropanol for 15 min before use.

## Note S2 Instrument characterization

UV-vis spectra were measured using a Shimadzu UV-2600i double-beam spectrophotometer. Electrochemistry experiments were performed using a Bio-logic electrochemical workstation at room temperature unless other mentioned. The morphology was observed with a Hitachi SU8200 scanning electron microscope (SEM), Bruker DIMENSION Icon (AFM), and Talos F200X Transmission Electron Microscope (TEM). Thermogravimetric analysis (TGA) was determined with a TGA209F1 (NETZSCH) at a heating rate of 10 ^o^C/min under a flow of nitrogen. X-ray diffraction (XRD) analysis was determined with ADVANCE D8 (Bruker). X-ray photoelectron spectroscopy (XPS) was performed using an AXIS SUPRA+. Fourier transform infrared (FT-IR) spectra were performed using a IS50 (Thermo Fisher Scientific).

Direct photolithography was performed with a UV LED lamp (centered at 365 nm, 60 mW/cm^2^) from Yanxizao Factory, Zhongshan, China. The power density was measured by UV-Integrator from Yeguan Factory, Shenzhen, China. EC materials were coated on the substrate by EZ4 SPIN COATER (Schwan technology).

## Note S3 Size calculation of WO_x_ nanoparticles

The size of WO_x_ nanoparticles was calculated using Scherrer equation (D = K λ / (β cosθ) (Ref S2). Here, K is a numerical factor frequently referred to as the crystallite-shape factor (~0.89). λ is the wavelength of the X-rays. β is the width (full-width at half-maximum) of the X-ray diffraction peak in radians and θ is the Bragg angle. And D is the crystallite size. Here, the clear XRD peak at 23.7 (2θ) was used to calculate the size of WO_x_ nanoparticles.

## Note S4 Adhesion test

The adhesion of WO_x_ films with ITO electrode was measured by the coat tester (EDKORS QFH-A). And a transparent tape (3M-600) was used to assist in peeling, as shown in Fig. S7.

Based on the international cross-cut method of Standard Test Methods for Rating Adhesion by Tape Test in ASTM-3359, the classification represents different film removal percent including 5B (None), 4B (less than 5%), 3B (5% - 15%), 2B (15% - 35%), 1B (35% - 65%) and 0B (greater than 65%).

# Supplementary Figures


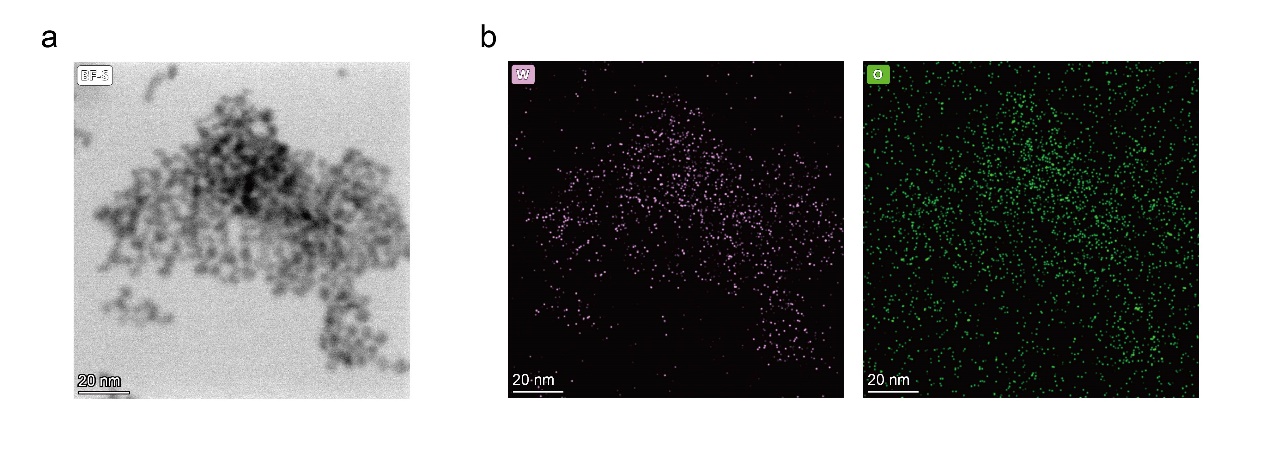


**Fig. S1** STEM image (**a**) and corresponding EDS elemental mapping of W and O elements (**b**)


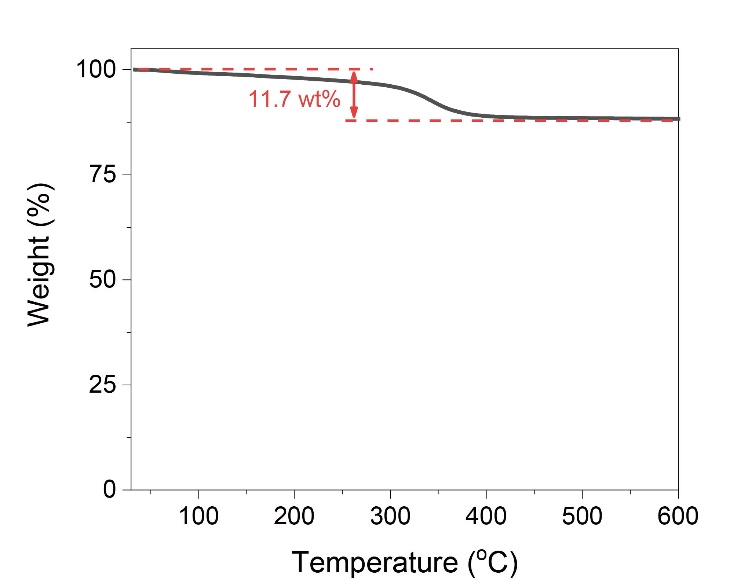


**Fig. S2** Result of thermogravimetry of as-synthesized WO_x_ NPs

Note: the content of surface ligand might be slightly lower than the actual value due to the loss of ligand during multiple post-processing and drying preparation for the test sample.


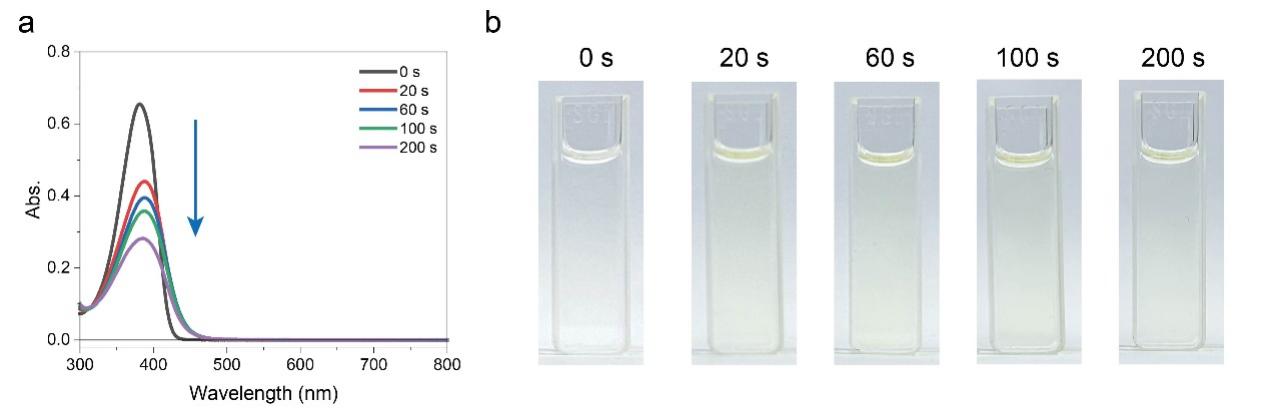


**Fig. S3** UV-vis spectra (**a**) and corresponding photos (**b**) of MBT (0.01 mg/mL in toluene) under a UV radiation (centered at 365 nm) for different times


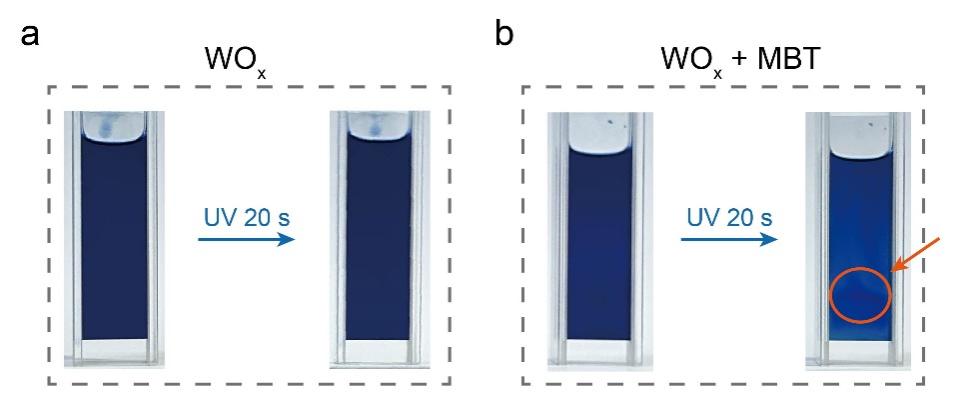


**Fig. S4** Photos of WO_x_ NPs (3 mg/mL in toluene) without (**a**) and with (**b**) 10 wt% MBT before and after UV radiation (centered at 365 nm). The insoluble precipitations were marked


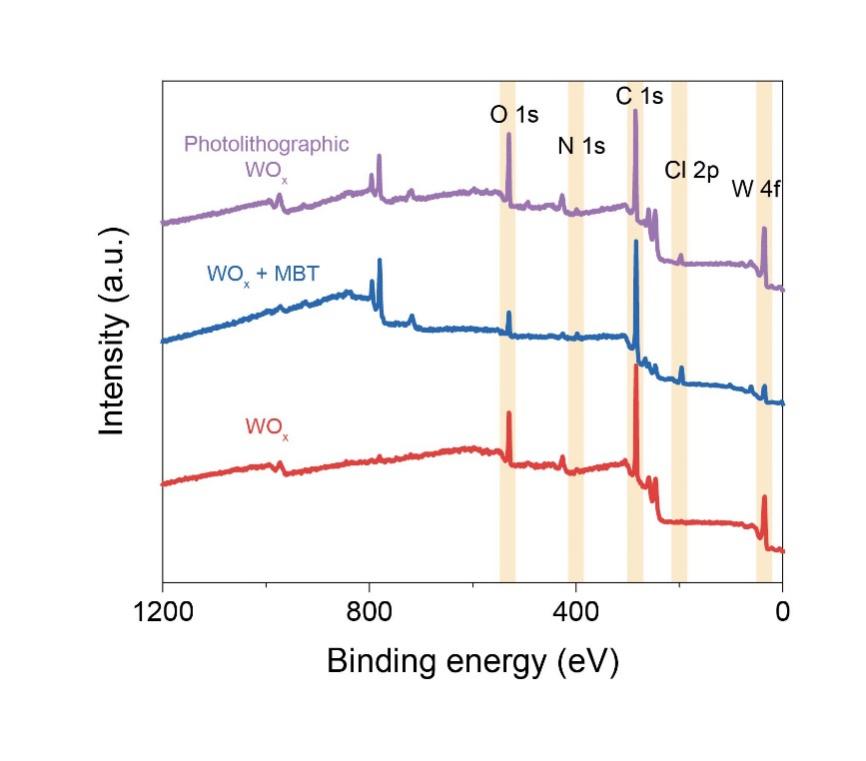


**Fig. S5** XPS survey spectra of pristine WO_x_, WO_x_ with 20.0 wt% MBT, and photolithographic WO_x_


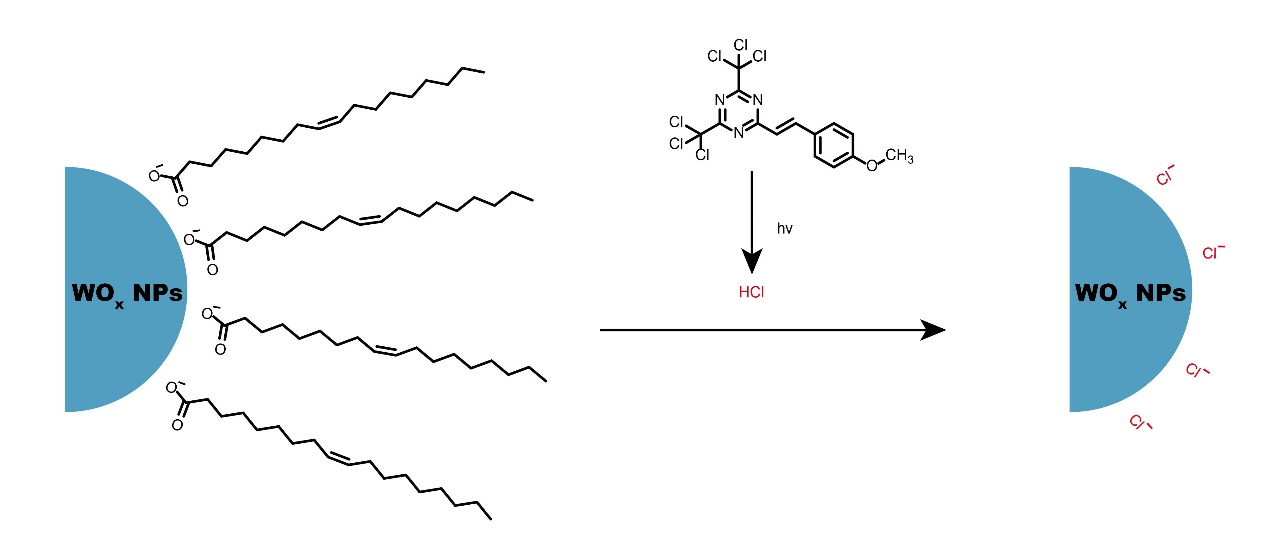


**Fig. S6** The schematic of direct photolithography of WO_x_ NPs *via* *in situ* ligand exchange from organic ligands, taking oleic acid (OA) as an example, to chlorine (Cl) ligand


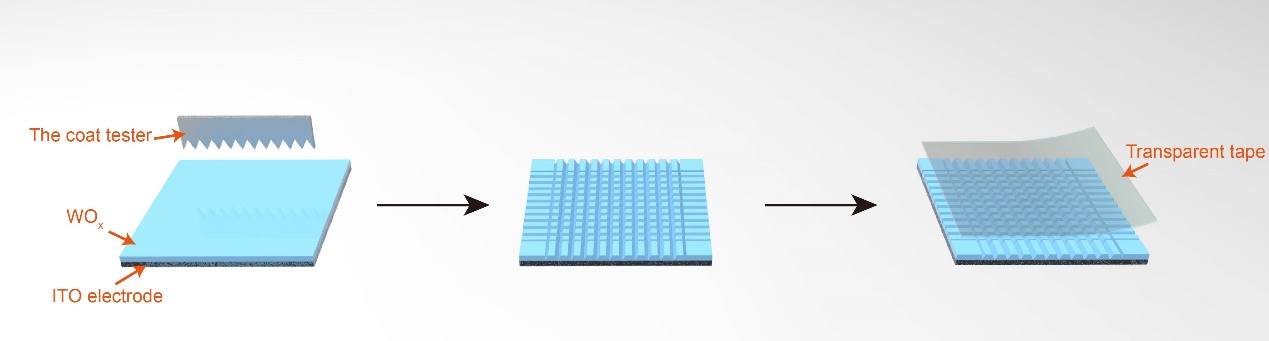


**Fig. S7** The schematic of the adhesion test of WO_x_ films on ITO electrode by the coat tester


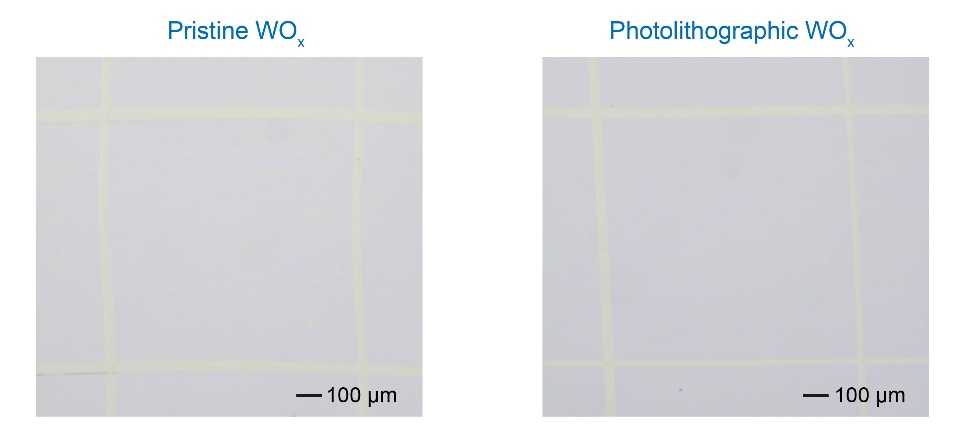


**Fig. S8** The microscopic images of pristine WO_x_ and photolithographic WO_x_ films treated by the coat tester


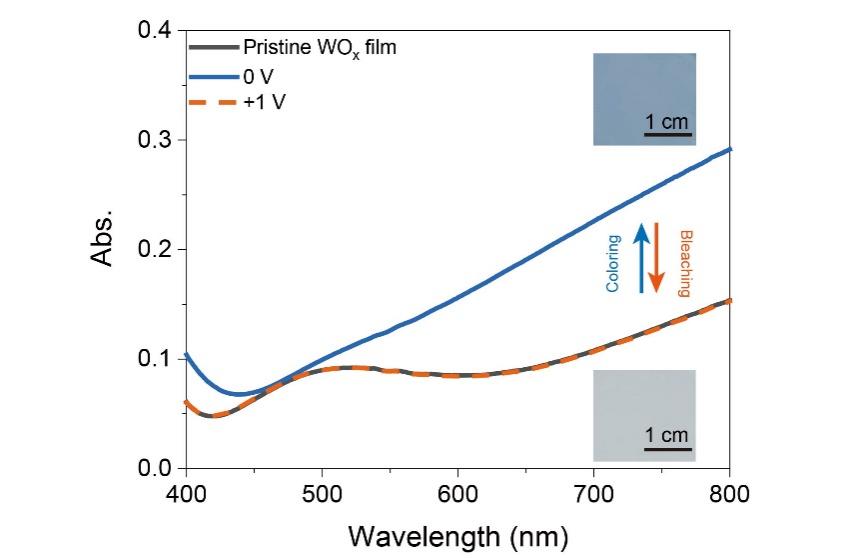


**Fig. S9** The absorption spectra in visible region of pristine WO_x_ film under different electrochemical stimulations and corresponding photos


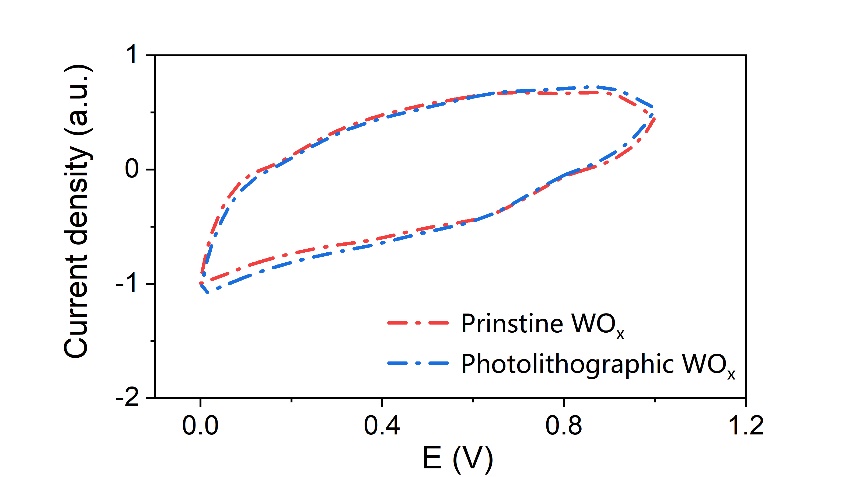


**Fig. S10** The CV data of pristine WO_x_ and photolithographic WO_x_. Scan rate: 100 mV/s. A zinc foil was used as the counter electrode. 1.0 mol/L ZnSO_4_ in deionized water was used as the electrolyte solution


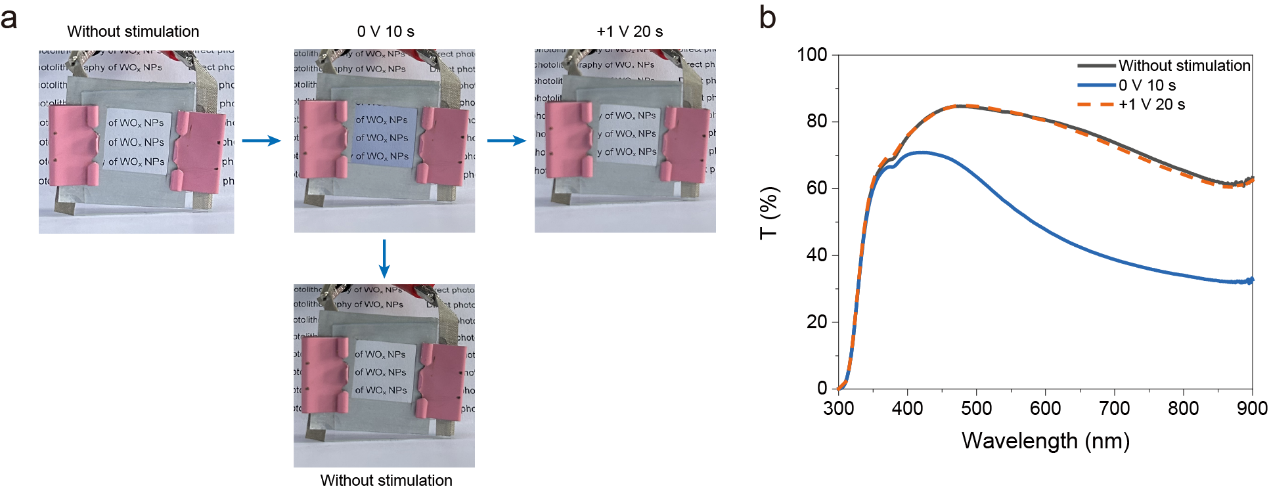


**Fig. S11** Photos (**a**) and transmittance spectra (**b**) of the self-powered EC device prepared by photolithographic WO_x_ NPs in different optical states


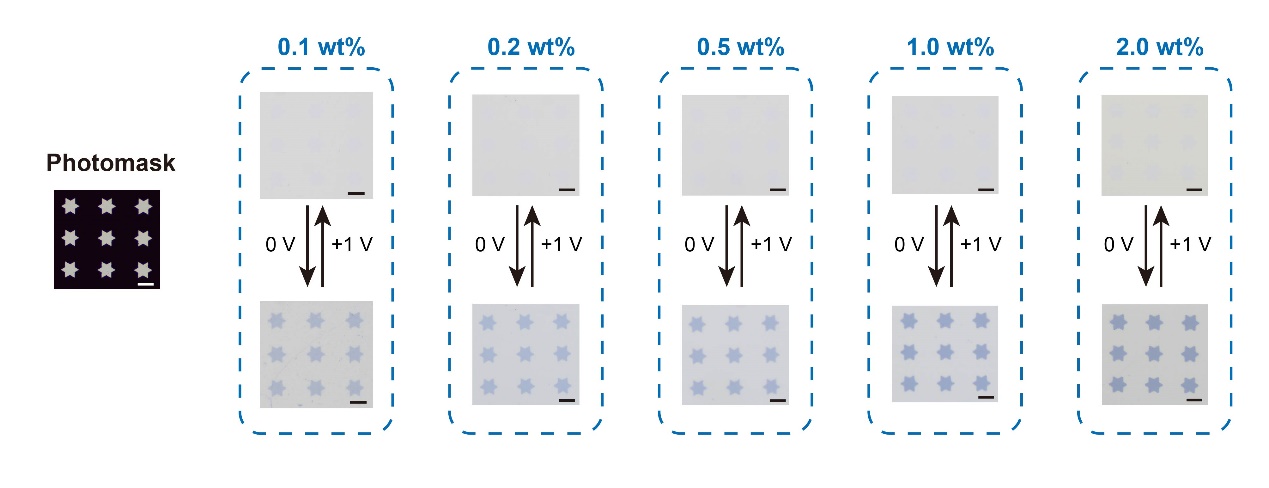


**Fig. S12** Photos of the photomask (left) and as-prepared EC devices (right) under different optical states with different MBT contents. Scale bar: 50 μm


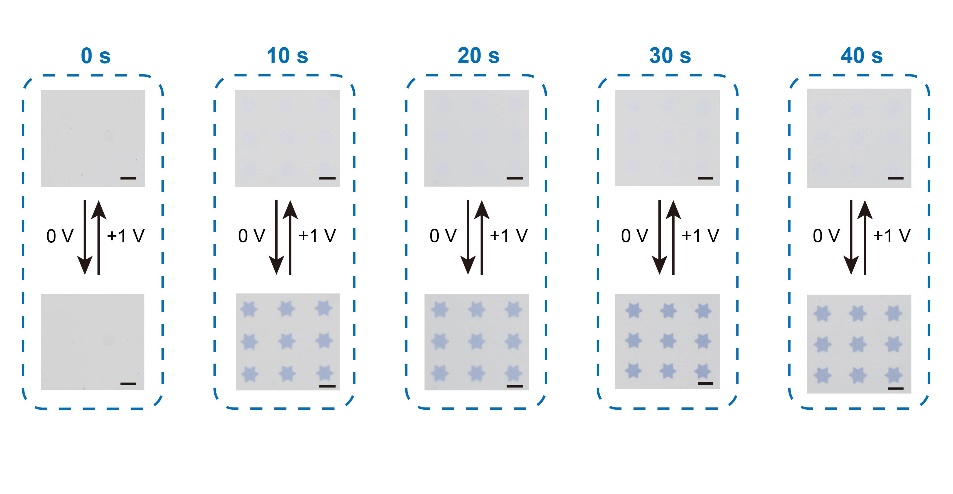


**Fig. S13** Photos of as-prepared EC devices under different optical states with different UV times. Scale bar: 50 μm


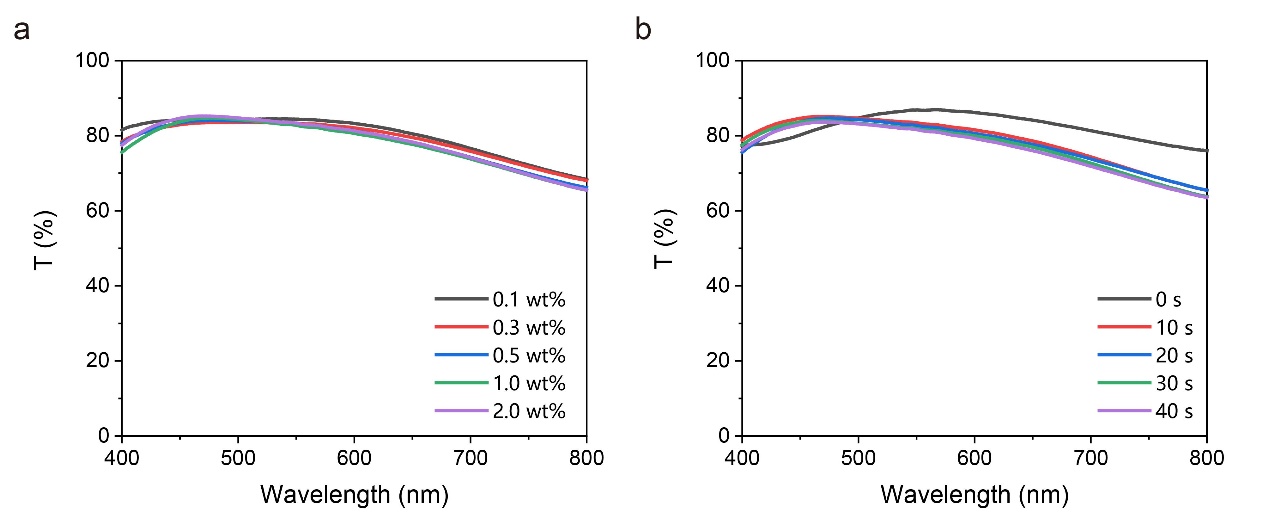


**Fig. S14** The visible spectra of EC devices based on photolithographic WO_x_ film with different MBT contents (**a**) and UV times (**b**). Note that the WO_x_ film could be dissolved by the developer when the UV time was 0 s, resulting a large spectral difference with other devices


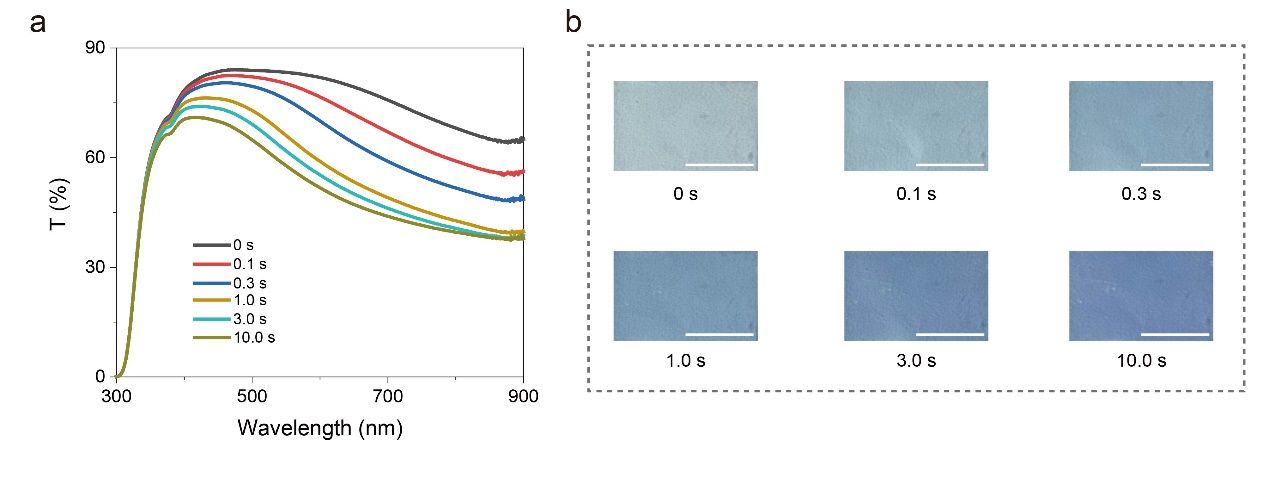


**Fig. S15** The UV-vis spectra (**a**) and photos (**b**) of the EC device based on photolithographic WO_x_ film (thickness: ~53 nm) under electrical stimulations of 0 V for different times. Scale bar: 1 cm


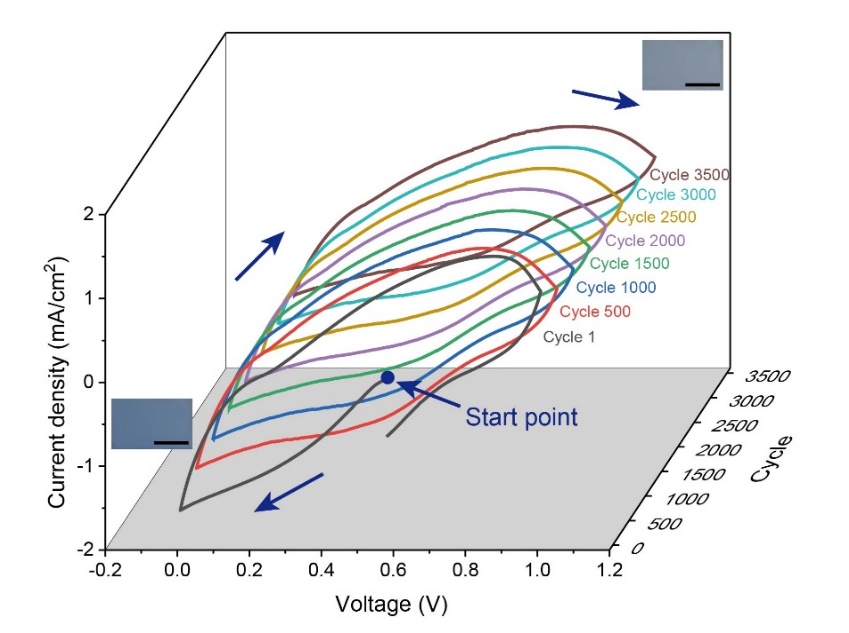


**Fig. S16** The CV data of the device at different cycles. Scan rate: 500 mV/s. Insert: photos of the device at the colored state and colorless state in CV tests. Scale bar: 1 cm


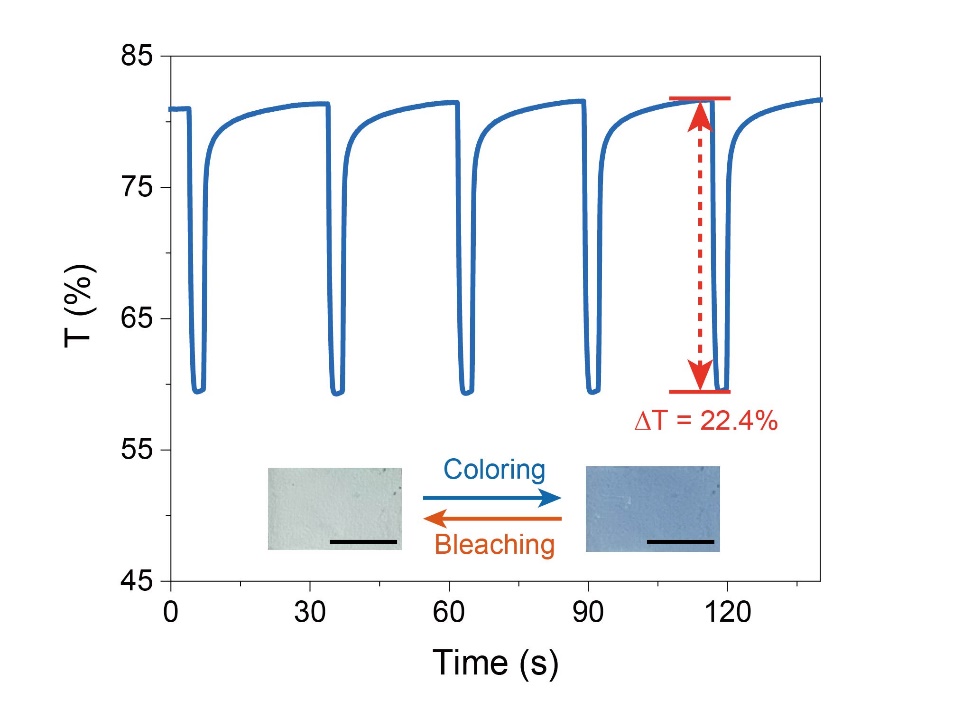


**Fig. S17** Transmittance at 633 nm of the EC device after cyclic stability test under the electrical stimulations of 0 V 1 s in coloring processes. And +1 V was used for corresponding bleaching processes. Insert: photos of the device at the colored state and colorless state. Scale bar: 1 cm.

Note: the device still had a remarkable optical modulation after cyclic stability test (ΔT = 22.4%). Compared with its optical modulation before cyclic stability test (ΔT = 28.7%), its optical modulation was well maintained (maintain ratio = ~78%).


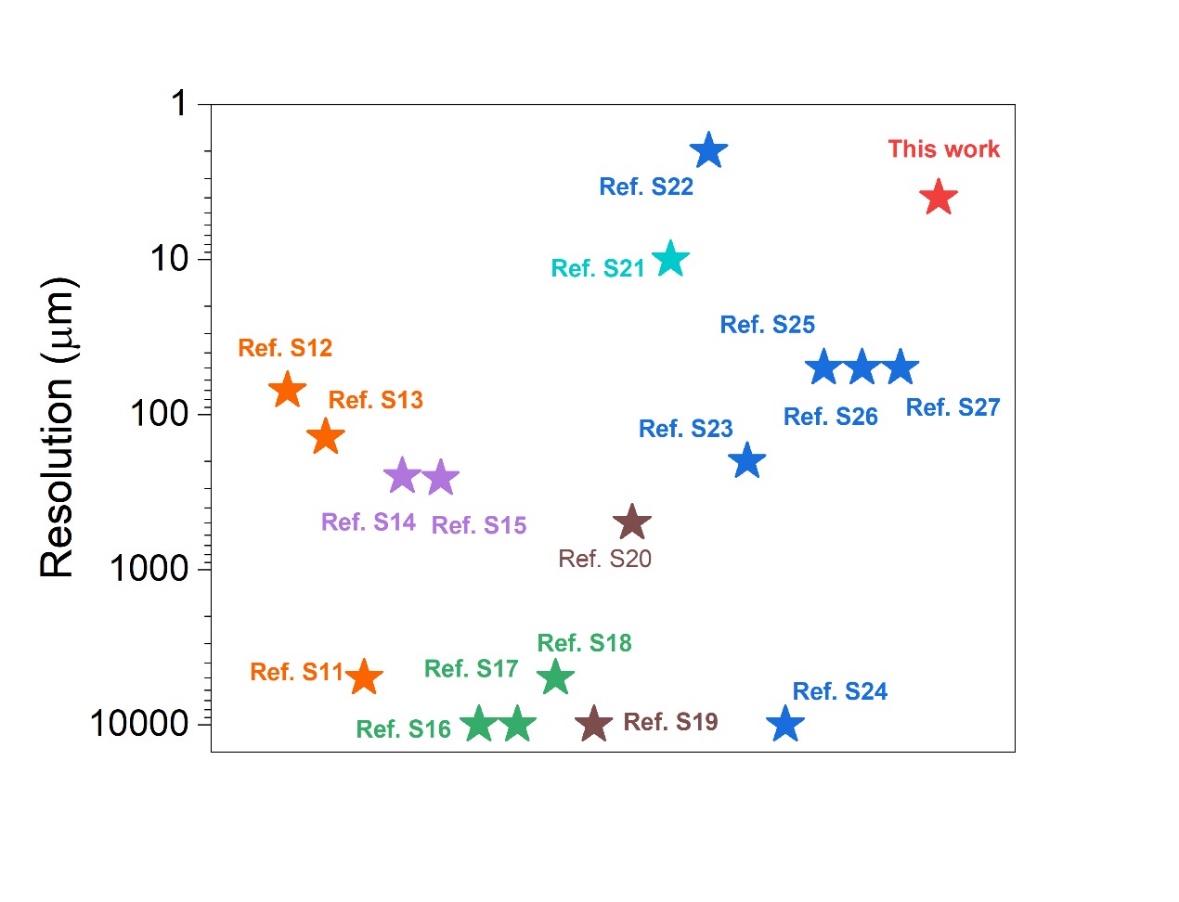


**Fig. S18** Reported resolution (pattern size or line width) of EC patterns. Corresponding data were listed on Table S3


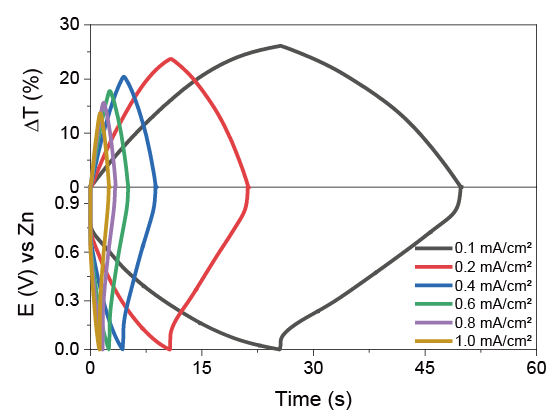


**Fig. S19** Galvanostatic charge and discharge curves (bottom) of EC devices based on photolithographic WO_x_ film and corresponding transmittance changes at 633 nm (top)


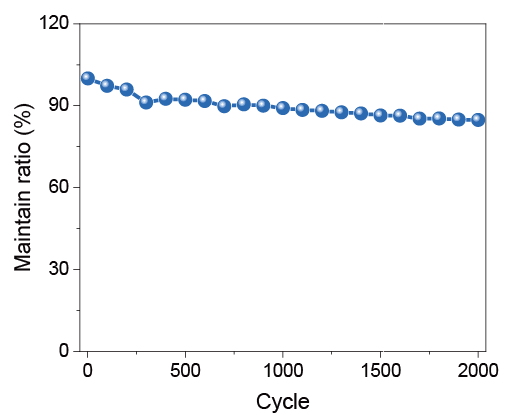


**Fig. S20** Long-term cycling performance at 1.0 mA/cm^2^ between 0 V and +1 V for 2000 cycles


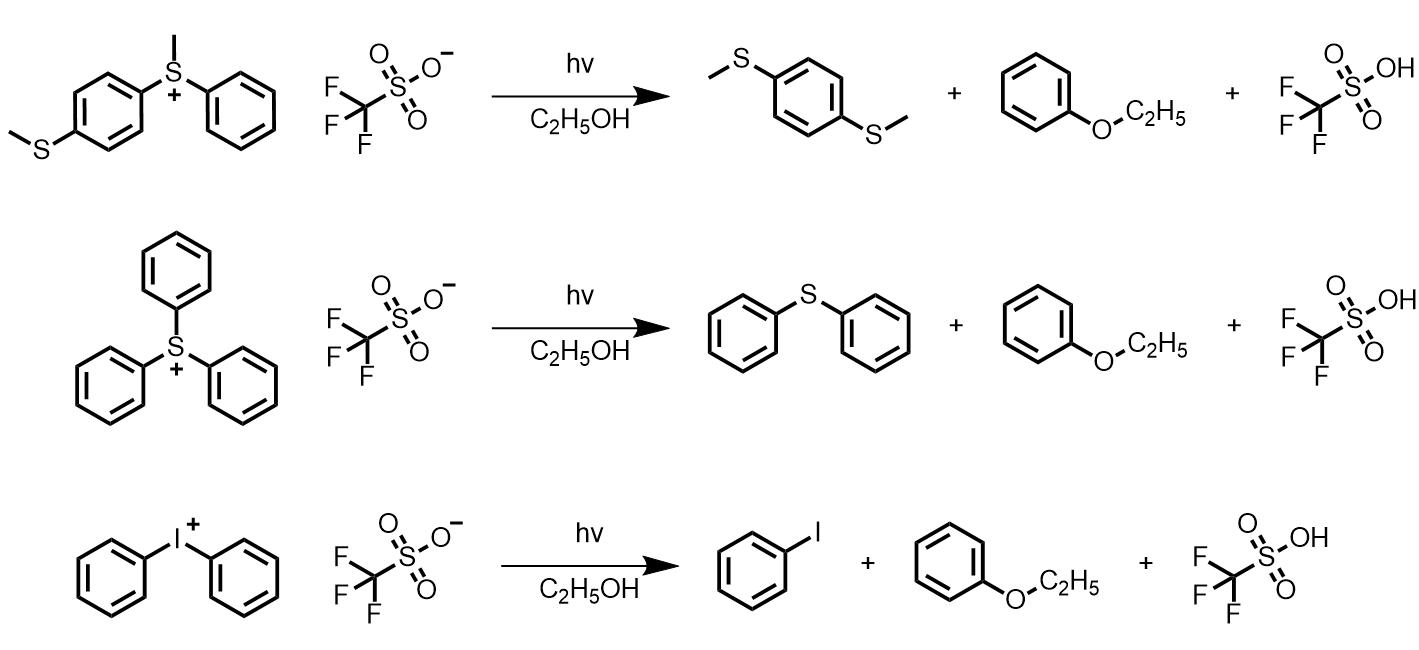


**Fig. S21** Photochemical reaction processes of PAGs under deep UV radiation

# Supplementary Tables

**Table S1** The thickness of photolithographic WO_x_ film *via* different spin speeds

| **Spin speed (rpm)** | **Thickness (nm)** |
| --- | --- |
| 5000 | ~18 |
| 3000 | ~30 |
| 1000 | ~53 |
| 500 | ~71 |
| 200 | ~90 |

**Table S2** EC performance of devices or films based on WO_x_

| **Refs.** | **EC materials** | **EC electrode or device** | **Coloring voltage (V)** | **Response**  **Speed (s)** | **Cyclic**  **stability** | **Optical**  **Modulation (%)** | **Coloration**  **efficiency**  **(cm^2^/C)** |
| --- | --- | --- | --- | --- | --- | --- | --- |
| [S1] | WO_x_ | Device | 0 V | 4.5 s | 1000 | 76% at 633 nm | 97.7 |
| [S3] | WO_3_ | Device | −3.0 V | 2.4 s | 2000 | <20% (reflection mode) | - |
| [S4] | WO_3_ | Device | +0.1 V | 4.0 s | 1000 | 72.6% | 101.6 |
| [S5] | WO_3_ | Device | 2.4 V | 0.7 s | 3000 | 90% at 650 nm | 109 |
| [S6] | WO_3_ | Device | 1.2 V | 5.7 s | 200 | ~77% | - |
| [S7] | m-WO_3-x_ | Film | −0.9 V (vs. Ag/Ag^+^) | 16 s | 2000 | 93.2% | 121 |
| [S8] | a-WO_3_ | Device | −3.5 V | 12 s | 1000 | 70% | 133 |
| [S9] | WO_3_ and NiO  nanostructured  films | Device | −2.5 V | 10 s | 100 | 75.4% | 131.9 |
| [S10] | WO_3_ | Electrode | 2.0 - 4.0 V (vs. Li/Li^+^) | - | 400 | 50% | - |
| [S11] | WO_3_ | Electrode | −1.2 V | - | ~8 | ~60% | 65.5 |
| ***This work*** | **WO_x_** | **Device** | **0 V** | **< 1 s** | **>3600** | **55.9% at 633 nm** | **119.5** |

**Table S3** Reported resolution of EC patterns

| **Refs.** | **Patterning method** | **EC materials** | **Pattern size or line width** |
| --- | --- | --- | --- |
| [S12] | Inkjet printing | Metallo-supramolecular polymers | 70 μm |
| [S13] |  | Conjugated polymer | 140 μm |
| [S11] |  | WO_3_ | < 5 mm |
| [S14] | Dispensing printing | WO_3_ | < 250 μm |
| [S15] |  | Viologen | 258 μm |
| [S16] | Screen printing | Conjugated polymer | ≥ 1 cm^2^ |
| [S17] |  | Conjugated polymer | < 1 cm^2^ |
| [S18] |  | Conjugated polymer | 5 × 5 mm^2^ |
| [S19] | Template | Viologen | 1 × 1 mm^2^ |
| [S20] |  | Conjugated polymer | 500 μm |
| [S21] | Thermal curing | Conjugated polymer | ≥ 10 μm |
| [S22] | Direct photolithography | Organic materials based on proton-coupled electron transfer | ~ 2 μm |
| [S23] |  | Viologen | 200 μm |
| [S24] |  | Conjugated polymer | 1 × 1 mm^2^ |
| [S25] |  | Conjugated polymer | 50 μm |
| [S26] |  | PProDOT (conjugated polymer) | < 50 μm |
| [S27] |  | PEDOT:PSS | < 50 μm |
| ***This work*** |  | **WO_x_ NPs** | **< 4 μm** |

# Supplementary References

1. L. Zhang, D. Chao, P. Yang, L. Weber, J. Li et al., Flexible pseudocapacitive electrochromics *via* inkjet printing of additive-free tungsten oxide nanocrystal ink. Adv. Energy Mater. **10**, 2000142 (2020). <https://doi.org/10.1002/aenm.202000142>
2. U. Holzwarth, N. Gibson, The scherrer equation versus the ‘Debye-scherrer equation’. Nat. Nanotechnol. **6**, 534 (2011). <https://doi.org/10.1038/nnano.2011.145>
3. Y. Li, P. Sun, J. Chen, X. Zha, X. Tang et al., Colorful electrochromic displays with high visual quality based on porous metamaterials. Adv. Mater. **35**, 2300116 (2023). <https://doi.org/10.1002/adma.202300116>
4. W. Zhang, H. Li, A.Y. Elezzabi A dual-mode electrochromic platform integrating zinc anode-based and rocking-chair electrochromic devices. Adv. Funct. Mater. **33**, 2300155 (2023). <https://doi.org/10.1002/adfm.202300155>
5. Z. Shao, A. Huang, C. Ming, J. Bell, P. Yu et al., All-solid-state proton-based tandem structures for fast-switching electrochromic devices. Nat. Electron. **5**, 45–52 (2022). <https://doi.org/10.1038/s41928-021-00697-4>
6. H. Li, C.J. Firby, A.Y. Elezzabi, Rechargeable aqueous hybrid Zn^2+^/Al^3+^ electrochromic batteries. Joule **3**, 2268–2278 (2019). <https://doi.org/10.1016/j.joule.2019.06.021>
7. S. Zhang, S. Cao, T. Zhang, A. Fisher, J.Y. Lee, Al^3+^ intercalation/de-intercalation-enabled dual-band electrochromic smart windows with a high optical modulation, quick response and long cycle life. Energy Environ. Sci. **11**, 2884–2892 (2018). <https://doi.org/10.1039/C8EE01718B>
8. W. Cheng, J. He, K.E. Dettelbach, N.J.J. Johnson, R.S. Sherbo et al., Photodeposited amorphous oxide films for electrochromic windows. Chem **4**, 821–832 (2018). <https://doi.org/10.1016/j.chempr.2017.12.030>
9. G. Cai, P. Darmawan, M. Cui, J. Chen, X. Wang et al., Inkjet-printed all solid-state electrochromic devices based on NiO/WO_3_ nanoparticle complementary electrodes. Nanoscale **8**, 348–357 (2016). <https://doi.org/10.1039/C5NR06995E>
10. R.-T. Wen, C.G. Granqvist, G.A. Niklasson, Eliminating degradation and uncovering ion-trapping dynamics in electrochromic WO_3_ thin films. Nat. Mater. **14**, 996–1001 (2015). <https://doi.org/10.1038/nmat4368>
11. M. Layani, P. Darmawan, W.L. Foo, L. Liu, A. Kamyshny et al., Nanostructured electrochromic films by inkjet printing on large area and flexible transparent silver electrodes. Nanoscale **6**, 4572–4576 (2014). <https://doi.org/10.1039/C3NR06890K>
12. B.-H. Chen, S.-Y. Kao, C.-W. Hu, M. Higuchi, K.-C. Ho et al., Printed multicolor high-contrast electrochromic devices. ACS Appl. Mater. Interfaces **7**, 25069–25076 (2015). <https://doi.org/10.1021/acsami.5b08061>
13. A.M. Österholm, D.E. Shen, D.S. Gottfried, J.R. Reynolds, Full color control and high-resolution patterning from inkjet printable cyan/magenta/yellow colored-to-colorless electrochromic polymer inks. Adv. Mater. Technol. **1**, 1600063 (2016). <https://doi.org/10.1002/admt.201600063>
14. X. Li, T.Y. Yun, K.W. Kim, S.H. Kim, H.C. Moon, Voltage-tunable dual image of electrostatic force-assisted dispensing printed, tungsten trioxide-based electrochromic devices with a symmetric configuration. ACS Appl. Mater. Interfaces **12**, 4022–4030 (2020). <https://doi.org/10.1021/acsami.9b21254>
15. K.W. Kim, H. Oh, J.H. Bae, H. Kim, H.C. Moon et al., Electrostatic-force-assisted dispensing printing of electrochromic gels for low-voltage displays. ACS Appl. Mater. Interfaces **9**, 18994–19000 (2017). <https://doi.org/10.1021/acsami.7b00946>
16. P. Andersson, R. Forchheimer, P. Tehrani, M. Berggren, Printable all-organic electrochromic active-matrix displays. Adv. Funct. Mater. **17**, 3074–3082 (2007). <https://doi.org/10.1002/adfm.200601241>
17. X. Cao, C. Lau, Y. Liu, F. Wu, H. Gui et al., Fully screen-printed, large-area, and flexible active-matrix electrochromic displays using carbon nanotube thin-film transistors. ACS Nano **10**, 9816–9822 (2016). <https://doi.org/10.1021/acsnano.6b05368>
18. P. Andersson Ersman, R. Lassnig, J. Strandberg, P. Dyreklev, Flexible active matrix addressed displays manufactured by screen printing. Adv. Eng. Mater. **23**, 2000771 (2021). <https://doi.org/10.1002/adem.202000771>
19. H.C. Moon, T.P. Lodge, C.D. Frisbie, Solution processable, electrochromic ion gels for sub-1 V, flexible displays on plastic. Chem. Mater. **27**, 1420–1425 (2015). <https://doi.org/10.1021/acs.chemmater.5b00026>
20. D. Kim, J. Kim, Y. Ko, K. Shim, J.H. Kim et al., A facile approach for constructing conductive polymer patterns for application in electrochromic devices and flexible microelectrodes. ACS Appl. Mater. Interfaces **8**, 33175–33182 (2016). <https://doi.org/10.1021/acsami.6b10103>
21. T.J. Gordon, J. Yu, C. Yang, S. Holdcroft, Direct thermal patterning of a π-conjugated polymer. Chem. Mater. **19**, 2155–2161 (2007). <https://doi.org/10.1021/cm062107c>
22. C. Gu, S. Wang, J. He, Y.-M. Zhang, S.X.-A. Zhang, High-durability organic electrochromic devices based on in-situ-photocurable electrochromic materials. Chem **9**, 2841–2854 (2023). <https://doi.org/10.1016/j.chempr.2023.05.015>
23. J.-W. Kim, J.-M. Myoung, Flexible and transparent electrochromic displays with simultaneously implementable subpixelated ion gel-based viologens by multiple patterning. Adv. Funct. Mater. **29**, 1808911 (2019). <https://doi.org/10.1002/adfm.201808911>
24. J. Jensen, A.L. Dyer, D.E. Shen, F.C. Krebs, J.R. Reynolds, Direct photopatterning of electrochromic polymers. Adv. Funct. Mater. **23**, 3728–3737 (2013). <https://doi.org/10.1002/adfm.201203005>
25. J. Kim, J. You, B. Kim, T. Park, E. Kim, Solution processable and patternable poly(3, 4-alkylenedioxythiophene)s for large-area electrochromic films. Adv. Mater. **23**, 4168–4173 (2011). <https://doi.org/10.1002/adma.201101900>
26. C. Huang, Y.-Q.-Q. Yi, Z. Hu, S. Zhang, X. Wu et al., Photolithographically patterned and highly stable electrochromic displays enabled by a photo-assisted cross-linker. J. Mater. Chem. C **11**, 15591–15598 (2023). <https://doi.org/10.1039/D3TC03088A>
27. G. Yang, J. Fan, K. Zhang, C. Gu, J. Li et al., Electrochromic reflective displays based on *in situ* photo-crosslinked PEDOT: PSS patterns. Adv. Funct. Mater. **34**, 2314983 (2024). <https://doi.org/10.1002/adfm.202314983>
